# 

**Published:** 1881-07

**Authors:** 


### CONTENTS:

ART. I.—The Imperfections of the English Dentists' Act. By W. A. N. Catlin, F. R. C. S. ....97
ART. II.—Rapid Breathing as a Pain Obtunder in Surgery, etc., etc. By W. G. A. Bonwill, D. D. S. ....111
ART. III.—Bleaching Teeth. By James Truman, D. D. S. ....123
ART. IV.—Dentists Constitutionally Treating Patients ....130
American Dental Association ....133
National Dental Association ....134
Southern and North Carolina Dental Associations ....134
Alabama Dental Association ....135
American Dental Convention ....135

### EDITORIAL, ETC.

The Bonwill Electro-Magnetic Mallet ....135
Another Journal ....137
Endorsements ....139
Preparatory Education ....139
Therapy ....140

### MONTHLY SUMMARY.

The Preservation of the Teeth ....141
Food for Infants ....141
Salivary Calculus ....142
Apparent Insusceptibility to the Influence of Nitrous Oxide ....143
Peculiarities of Race ....144
Peculiar Obstruction of Wharton's Duct ....144

